# Comparative analysis of excretory-secretory antigens of *Trichinella spiralis *and *Trichinella britovi *muscle larvae by two-dimensional difference gel electrophoresis and immunoblotting

**DOI:** 10.1186/1477-5956-10-10

**Published:** 2012-02-11

**Authors:** Justyna Bien, Anu Näreaho, Pekka Varmanen, Katarzyna Gozdzik, Bozena Moskwa, Wladyslaw Cabaj, Tuula A Nyman, Kirsi Savijoki

**Affiliations:** 1 Witold Stefanski Institute of Parasitology of the Polish Academy of Sciences, 51/55 Twarda Street, 00818 Warsaw, Poland; 2 Department of Veterinary Biosciences, University of Helsinki, P.O. Box 66, 00014 Helsinki, Finland; 3 Department of Food and Environmental Sciences, University of Helsinki, P.O. Box 66, 00014 Helsinki, Finland; 4 Institute of Biotechnology, University of Helsinki, P.O. Box 65, 00014 Helsinki, Finland

**Keywords:** 2-D DIGE, E-S, *Trichinella spiralis*, *Trichinella britovi*, Immunoblotting

## Abstract

**Background:**

Trichinellosis is a zoonotic disease in humans caused by *Trichinella *spp. The present study was undertaken to discover excretory-secretory (E-S) proteins from *T. spiralis *and *T. britovi *muscle larvae (ML) that hold promise for species-specific diagnostics. To that end, the purified E-S proteins were analyzed by fluorescent two-dimensional difference gel electrophoresis (2-D DIGE) coupled with protein identification by liquid chromatography-tandem mass spectrometry (LC-MS/MS). To search for immunoreactive proteins that are specifically recognized by host antibodies the E-S proteins were subjected to two-dimensional (2-DE) immunoblotting with antisera derived from pigs experimentally infected with *T. spiralis *or *T. britovi*.

**Results:**

According to 2-D DIGE analysis, a total of twenty-two proteins including potentially immunogenic proteins and proteins produced only by one of the two *Trichinella *species were subjected to LC-MS/MS for protein identification. From these proteins seventeen could be identified, of which many were identified in multiple spots, suggesting that they have undergone post-translational modification, possibly involving glycosylation and/or proteolysis. These proteins included 5'-nucleotidase, serine-type protease/proteinase, and p43 glycoprotein (gp43) as well as 49 kDa E-S protein (p49). Our findings also suggest that some of the commonly identified proteins were post-translationally modified to different extents, which in certain cases seemed to result in species-specific modification. Both commonly and specifically recognized immunoreactive proteins were identified by 2-DE immunoblotting; shared antigens were identified as gp43 and different protease variants, whereas those specific to *T. britovi *included multiple isoforms of the 5'-nucleotidase.

**Conclusions:**

Both 2-D DIGE and 2-DE immunoblotting approaches indicate that *T. spiralis *and *T. britovi *produce somewhat distinctive antigen profiles, which contain E-S antigens with potential as species-specific diagnostic markers for *Trichinella*. Our results also demonstrate the value of 2-D DIGE as a versatile tool to compare secretomes of different *Trichinella *species for pinpointing factors contributing to the interaction with the host.

## Background

Trichinellosis is a food-borne parasitic zoonosis caused by nematodes of the genus *Trichinella*. Twelve genotypes of *Trichinella *have been identified worldwide [1-3], four of which are confirmed to exist in Europe: *T. spiralis, T. nativa, T. britovi *and *T. pseudospiralis*. In Poland, during various epidemiological surveys, only two *Trichinella *species have been identified in domestic and wild animals, *T. spiralis *and *T. britovi *[4,5]. *T. spiralis *is the etiological agent of most human infections and deaths caused by trichinellosis globally, although other encapsulating and nonencapsulating species can cause human infections, including *T. britovi*, *T. nativa*, and *T. pseudospiralis *[6-11]. Trichinellosis is mainly acquired by the ingestion of domestic animal meat, such as pig and horse meat, that contains infective larvae. In addition to domestic sources of infection, sylvatic transmission via the consumption of wild game is also an important source of human infection [6,7,12]. Mixed *Trichinella *species infections appear to be a common phenomenon, and have been reported in several host species [13-18].

The life-cycle of *Trichinella *spp. begins with the consumption of meat that contains infective muscle larvae (ML). In the host stomach, larvae are released into gastric fluid and develop into adult worms (females and males) in the host intestine, and the female begins to release the newborn larvae (NBL). The NBL penetrate the intestinal wall, enter the lymphatic system, and migrate through the bloodstream into striated muscle where it infects and encapsulates within a portion of the myofiber and develops into the infective muscle larvae. During this process an intimate host-parasite interaction is formed [19]. *Trichinella *spp. are believed to modulate host cell functions for their own benefit, and in this process the excretory-secretory (E-S) proteins produced by muscle larvae are believed to play a crucial role [20].

Although clinical differences have been observed among people infected with different species of *Trichinella*, it has not been possible to attribute these differences to the species of the pathogen because the number of infecting larvae ingested by each person was generally unknown [21]. The clinical and biological features observed during human infection with *T. spiralis *appear to have been different from those caused due to *T. britovi*. The main distinctions between the two types of infections were a longer duration of parasite-specific IgG, increased creatine phosphokinase (CPK) levels, and a more severe intestinal symptomatology in *T. spiralis*-infected patients than in those infected with *T. britovi*, and this could be due to the fact that *T. britovi *females are less profilic [22]. *T. murrelli *seems to be more likely to provoke skin reactions and facial oedema [23]. *T. pseudospiralis*, which is non-encapsulated, seems to provoke signs and symptoms that last longer [24,25].

Up to now, it has not been possible to differentiate *Trichinella *species serologically, although several methods including ELISA, Western Blot, immunochemical and immunoprecipitation assays are widely used for the diagnosis of *Trichinella *in humans [21,26-30]. Serological methods are not particularly appropriate for early and species-specific diagnostics. Nevertheless, diagnostics could be greatly improved if the infecting species can be identified serologically. Therefore, information about E-S antigens, that are common or unique to different *Trichinella *species, is required to aid the development of species-specific diagnostics. A genome sequence, enabling all potential E-S antigens in *T. spiralis *to be pinpointed, was recently completed [31]. Nonetheless, as the genetic information is only indicative of the cell's potential, proteome-wide analysis is needed to complement the genetic information.

Fluorescent two-dimensional difference gel electrophoresis (2-D DIGE) is the latest multiplexed innovation in two-dimensional gel electrophoresis (2-DE), which incorporates distinctive fluorescent dyes, and enables detection of relative protein abundance differences with over a 10,000-fold protein concentration range [32-34]. Protein analysis based on 2-DE is also the method of choice to demonstrate post-translational modifications involving, for example, glycosylation, phosporylation or proteolysis. The proteomic studies reported for *Trichinella *were based on the application of the classical 2-DE and have been highly effective in the characterization of the proteomes of different species of *Trichinella*, including *T. spiralis, T. britovi, T. pseudospiralis, T. nativa *and T8 [35-39].

In the current study we applied 2-D DIGE technique and 2-DE immunoblotting to uncover common and unique E-S proteins produced by *T. spiralis *and *T. britovi *muscle larvae.

## Methods

### Parasites

*Trichinella spiralis *(strain ISS-003) and *T. britovi *(strain ISS-002) ML (Istituto Superiore di Sanita, The International *Trichinella *Reference Centre) were maintained in female Balb/c mice. ML of both species were isolated from infected mice as previously described [40,41].

### Collection and preparation of E-S proteins

Larvae of *T. spiralis *and *T. britovi *were washed several times in pre-warmed RPMI 1640 medium (Sigma-Aldrich Chemie GmbH, Steinheim) and resuspended at 5000 larvae ml^-1 ^in RPMI containing 2 mM L-glutamine, and antibiotics (100 U ml^-1 ^penicillin, 100 μg ml^-1 ^streptomycin). ML were incubated with 5% CO_2 _in a 75 cm^2 ^culture flask at 37°C for up to 18 h, after which they were allowed to sediment by settling in 50 ml conical tubes. The culture supernatants containing the E-S proteins were filtered through a 0.22 μm filter and then concentrated by lyophilization.

### CyDye labeling of E-S proteins

The E-S extracts of *T. spiralis *and *T. britovi *were purified using a 2-D Clean-Up Kit (GE Healthcare) and solubilised in 10-20 μL of UTCT composed of 7 M urea (Sigma-Aldrich), 2 M thiourea (Sigma-Aldrich), 4% CHAPS (Sigma-Aldrich) and 30 mM Trizma base (Bio-Rad). The protein concentration was determined using a 2-D Quant Kit (GE Healthcare) according to the manufacturer's protocol. Prior to CyDye labelling, the pH of each protein sample was adjusted to 8.5 by the addition of UTCT. Both the *T. spiralis *and *T. britovi *protein samples were divided in four to represent technical replicate samples prior to CyDye labelling. The samples were then labelled using Cy2, Cy3, or Cy5 dyes (CyDye DIGE Fluor minimal dyes; GE Healthcare), according to the Ettan™ 2-D DIGE protocol. Briefly, approximately 12.5 μg of protein from *T. spiralis *and *T. britovi *was labelled with 200 pmol of the Cy3 and Cy5 dyes. As an internal standard, aliquots from each sample were combined and labelled with Cy2 dye. To exclude dye-specific effects, Cy3 and Cy5 were used interchangeably according to a dye-swapping approach (Table 1). The labelling mixtures were incubated on ice in the dark for 30 min and the reactions were quenched with 1 mM lysine (Sigma-Aldrich) followed by incubation on ice for 10 min. The labelled samples were pooled and separated by 2-DE as detailed below.

**Table 1:** Setup of a DIGE experiment using four technical repeats of E-S protein samples and a dye swap between Cy3 and Cy5

Gel number	Cy3	Cy5	Cy2
1	TS1^a^	TB1^a^	Mix^b^
2	TS2	TB2	Mix
3	TB3	TS3	Mix
4	TB4	TS4	Mix

^a^TS and TB refers to E-S protein samples concentrated and purified from *T. spiralis *and *T. britovi*, respectively

^b^Mix refer mixture of samples of all four repeats

### 2-DE and DeCyder analysis

CyDye labeled protein samples were separated by isoelectric focusing (IEF) using IPG strips (11 cm, NL, pH 3-10, Bio-Rad) and a Protean IEF Cell (Bio-Rad). The IEF strips were rehydrated overnight in 500 μL of the rehydration solution (GE Healthcare) containing 1% Bio-Lyte pH 3-10 (Bio-Rad). Samples containing approximately 50 μg protein in 5 mM DTT, 5 mM tributylphosphine, and 1% Bio-Lyte pH 3-10 were applied to the IPG strips by cup-loading. IEF was performed using a Protean IEF Cell at 20°C as follows: 15 min at 250 V, then linear ramping to 8000 V for 25,000 Vh, and 8000 V for 10,000 Vh (using a limit of 50 μA/strip).

After IEF the strips were first equilibrated for 25 min in a buffer containing 50 mM Tris-HCl (pH 6.8), 6 M urea, 2% SDS, and 20% glycerol and supplemented with 2% DTT (buffer A), followed by a 25 min equilibration in the same buffer in which DTT was replaced with 2.5% iodoacetamide (buffer B). The second dimension SDS-PAGE was run on Criterion PreCast gels (12.5% Tris-HCl gels, BioRad) in a Criterion Dodeca Cell (BioRad) with 200 V for approximately one hour.

The gels were scanned between low fluorescence glass plates using a FLA-5100 laser scanner (Fujifilm) at wavelengths of 473 nm (for Cy2), 532 nm (Cy3), and 635 nm (Cy5) using voltages of 420, 410, and 400 V, accordingly (with 100 μm resolution). The gel images were cropped to identical size by removing areas extraneous to the protein spots with ImageQuant TL 7.0 software (GE Healthcare). After scanning, the gels were fixed in 30% ethanol and 0.5% acetic acid for 60 min minimum and then silver stained [42]. Image and statistical analyses for the cropped 2-D DIGE gels were performed using DeCyder 2D 6.5 software (GE Healthcare). With the use of a batch processor function, the gels were first automatically analysed in a differential in-gel analysis (DIA) module, which normalized the Cy2, Cy3, and Cy5 image from each gel. Spot boundaries were detected, and spot volumes (protein abundances) were calculated. Then, the spot volumes of Cy3 and Cy5 samples were compared with the spot volumes of the Cy2 sample (internal standard) to generate standard spot volumes, thereby correcting intergel variations. In the biological variation analysis (BVA) module, the Cy2 images of four replicate gels were matched, and the standard spot volume ratios between all four gels were compared. The DeCyder BVA module was used to matching multiple 2-D DIGE gels for comparison and statistical analysis of protein abundance changes. Spots with at least a 1.2-fold spot volume ratio change and a *p *value of lower than 0.05 were selected for identification.

### LC-MS/MS analysis

Mass spectrometric (MS)-compatible silver staining [43] was performed to visualize the protein spots for identification. Protein spots of interest were in-gel digested with trypsin and the peptides recovered as previously described [44]. The resulting peptides were analyzed by fragment ion analysis with LC-MS/MS using an Ultimate 3000 nano-LC (Dionex, Sunnyvale, CA, USA) and QSTAR Elite hybrid quadrupole TOF mass spectrometer (Applied Biosystems/MDS Sciex, Foster City, CA, USA) with nano-ESI ionisation. The samples were first concentrated and desalted on a C_18 _trap column (10 mm × 150 μm, 3 μm, 120 Å, PROTECOL™; SGE Analytical Science, Griesheim, Germany) followed by peptide separation on a PepMap100 C_18 _analytical column (15 cm × 75 μm, 5 μm, 100 Å; LC Packings, Sunnyvale, CA, USA) at 200 nl/min. The separation gradient consisted of 0-50% B in 20 min, 50% B for 3 min, 50-100% B in 2 min and 100% B for 3 min (buffer A: 0.1% formic acid; buffer B: 0.08% formic acid in 80% acetonitrile). MS data were acquired using Analyst QS 2.0 software. Information-dependent acquisition method consisted of a 0.5 s TOF-MS survey scan of m/z 400-1400. From every survey scan two most abundant ions with charge states +2 to +4 were selected for product ion scans. Once an ion was selected for MS/MS fragmentation, it was put on an exclusion list for 60 s.

### Protein identification

All LC-MS/MS results were analyzed using the ProteinPilot™ (version 2.0.1, Applied Biosystems) software. The MS/MS data were searched against the NCBINr database 20111206 (16392747 sequences; 5641810382 residues) restricted to Metazoa (Metazoa (Animals) (2796749 sequences) and/or Other Metazoa (989730 sequences) using Mascot (Matrix Science, version 2.2.03). The search criteria for were: trypsin digestion with one missed cleavage allowed, carbamidomethyl modification of cysteine as a fixed modification and oxidation of methionine as variable modification. For the LC-MS/MS spectra both the maximum precursor ion mass tolerance and MS/MS fragment ion mass tolerance were 0.2 Da, and peptide charge state of +1, +2, +3 was used. A successful identification was reported when a significant match (*p *< 0.05) was obtained.

### 2-DE coupled with immunoblotting

For two-dimensional immunoblotting, rehydration of IPG strips (13 cm, NL, pH 3-10), IEF followed by 2-DE separation of *T. spiralis *and *T. britovi *E-S proteins (50 μg each) was performed as described above. After 2-DE, proteins were transferred to a polyvinylidene fluoride membrane (Bio-Rad) that was then exposed to sera from experimentally *T. spiralis *or *T. britovi *infected pigs (1:100), followed by goat anti-pig IgG conjugated to horse-radish peroxidase (1:20 000; Bethyl Laboratories. Inc). The blots were developed using the SuperSignal West Dura Extended Duration kit (Pierce) and visualized with a Fuji LAS3000 CCD camera (Fuji Photo Co. Ltd, Japan). The experiment was performed using two biological replicate samples.

## Results and Discussion

### 2D DIGE analysis of *T. spiralis *and *T. britovi *proteomes

**Figure 1:**
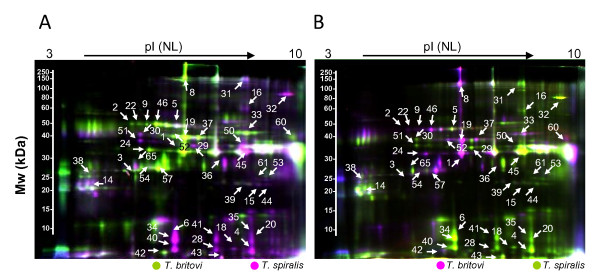
**A representative overlay image of fluorescent 2D-gels containing E-S proteins extracted from *T. spiralis *and *T. britovi***. The total amount of protein used for CyDye labeling was 50 μg. Protein spots appearing in purple or green refer to proteins that are more abundant in *T. spiralis *or in *T. britovi *(**A**) *T. spiralis *and *T. britovi *proteins labelled with Cy3 and Cy5, respectively. (**B**) *T. spiralis *and *T. britovi *proteins labelled with Cy5 and Cy3 respectively. Protein spots appearing in white represent E-S proteins from both species. The numbered protein spots, cut from the same gels post-stained with silver, were identified by LC-MS/MS and are listed in Additional file 1: Table S2.

**Figure 2:**
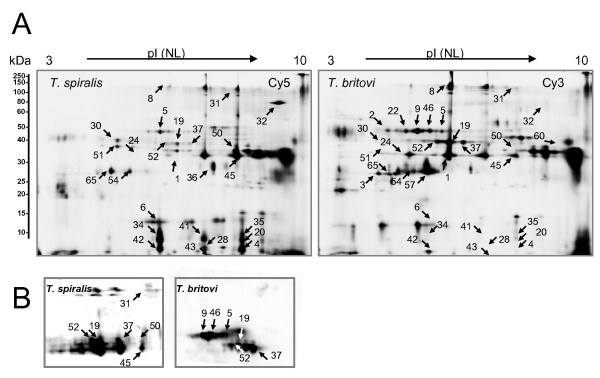
**(A) Representative Cy-labeled gel images illustrating the *T. spiralis *(Cy5) and *T. britovi *(Cy3) and E-S proteomes**. (**B**) 2D immunoblotting of *T. spiralis *and *T. britovi *E-S proteins with *T. spiralis *and *T. britovi *antisera. The numbered protein spots correspond to those identified from 2-D DIGE gels (Additional file 1: Table S2, Figure 1).

**Figure 3:**
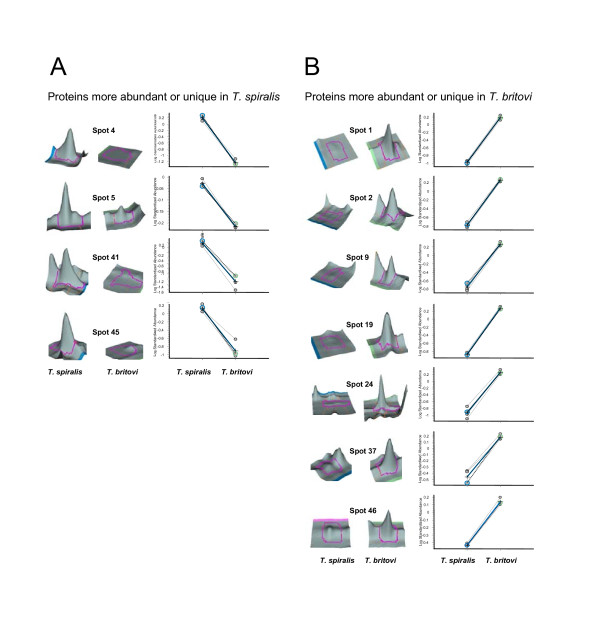
**A representative three-dimensional (3D) image of chosen DIGE spots that are more abundant or unique in *T. spiralis *(A) or *T. britovi *(B) 3D images and statistics were generated using the BVA module of the DeCyder software**.

The present study aimed to identify antigenic E-S proteins produced by *T. spiralis *and *T. britovi *which may elicit an immune response in infected pigs, and thus hold promise as potential target proteins for species-specific diagnostics. To meet this goal, we have previously conducted immunoblot analyses to estimate the size of the *T. spiralis *and *T. britovi *E-S proteins that cross-react with species-specific antisera (data not shown). To gain more knowledge of these proteins and to pinpoint which antigens are uniquely produced we wished to apply 2D DIGE that enables quantative comparison of the indicated E-S proteins in the same gel. For this purpose, four technical replicate samples (each containing 12.5 μg protein) were differentially labeled with CyDye labels and pooled for IEF and 2D separation using the experimental setup in Table 1. Co-detection of the scanned 2D gel images using DeCyder software enabled detection of 150-200 spot features in both *Trichinella *proteomes over the pI range of 3-10. Figures 1A, B and 2A show the fluorescent and the typical Cy5/Cy3 overlays of the *T. spiralis *and *T. britovi *specific E-S proteomes. The *T. spiralis *and *T. britovi *E-S proteomes are distinctly different (Figure 2A). After BVA analysis each proteome was shown to contain 44 proteins exhibiting differential abundance (> 1.2-fold spot volume ratio change and *p *< 0.05) or appearing only in one of the two proteomes. From these protein spots fourteen (spots 4, 6, 18, 20, 28, 32, 34, 35, 40, 41, 43, 50, 51 and 65) were found to be specifically produced by *T. spiralis*, whereas only eight proteins were uniquely produced by *T. britovi *(spots 2, 9, 19, 22, 24, 42, 57 and 60) (Figure 1A, B, 2A). A three-dimensional view of the 2-DE gel regions containing chosen species-specific protein spots further support the unique appearance of the proteins in *T. spiralis *and *T. britovi *E-S proteomes (Figure 3A, B). The remainder of the protein spots (22 in total), were expected to contain proteins that are produced in different amounts by *T. spiralis *and *T. britovi*, as evidenced for the selected protein spots in Figure 3A, B.

### Majority of the proteins were identified as having multiple charge isoforms

We have previously observed that the potentially immunogenic E-S proteins could migrate with Mw values between 30 and 50 kDa (data not shown). Thus, from all *T. spiralis *and *T. britovi *proteins showing different abundance we selected protein spots that migrate within this region for identification by LC-MS/MS. We also selected a subset of other proteins that seemed to be uniquely produced by either one of the two species to gain deeper insight into the mechanisms by which these two different species infect the host. Thus, in total of twenty-two protein spots were selected for final identification, and from these spots only seventeen proteins were successfully identified (Additional file 1: Table S2). In the *T. spiralis *E-S proteome, the specifically appearing protein spots were identified as a probable 5'nucleotidase, a glycoprotein p43 (gp43) and two potential serine proteases with different pI and molecular weight values (Additional file 1: Table S2, Figure 1A, B). The species-specific protein spots appearing only in the *T. britovi *E-S proteome were also found to contain a potential 5'nucleotidase, serine protease/proteinase and 49 kDa E-S antigen (p49), which all were identified in multiple protein spots (Additional file 1: Table S2; Figure 1A, B). There are a number of possible explanations for these different isoforms, including amino acid sequence differences, alternative splicing, or post-translational modifications. Similarly, Robinson and Connolly [36] identified 43 out of 52 E-S protein spots from *T. spiralis*, which represented only 13 different proteins indicating that there are multiple charge isoforms produced by this species. They observed that the most prominent proteins included the serine protease, the 45-kDa antigen, and the gp43 [36]. In our study, the variable glycosylation is one of the likely reasons explaining the identification of one and the same protein in several horizontally migrating spots (Figure 1A, B). This is supported by previous proteomic studies, which showed that the 5'-nucleotidase of *T. spiralis *contains three potential N-glycosylation sites [45], and that native p49 protein undergoes glycosylation [46-48]. Such modification is also known to affect the pI and Mw values of the protein [49], thereby explaining the unexpected migration pattern (i.e., increased p/Mw values) of the identified p49 antigen variants (spots 24 and 60) in *T. britovi *(Figure 1A, B, Additional file 1: Table S2). Similarly, previous studies done by Romaris et al. have also demonstrated that *T. spiralis *may express more than one isoform of the protein and that a common precursor protein could undergo variable post-translational processing [50]. Our findings support this as the serine-type proteases were also identified in several protein spots in *T. spiralis *E-S proteome (Figure 1A, B, Additional file 1: Table S2). Protein modification could also be the reason behind the unsuccessful MS-identification of four protein spots (spots 4, 6, 40, 41) that appear to be specific for *T. spiralis *(Figure 1A, B). No conclusive identification was obtained for these protein spots. The MS/MS spectra after LC-MS/MS analysis of the tryptic peptides derived from these proteins could not be matched with recently released predicted proteins from *T. spiralis *[31]. Additionally, several proteins specifically produced by *T. spiralis *had low molecular mass for which the LC-MS/MS analysis failed to provide positive identification. In addition, the distinct migration pattern of these proteins (proteins with the same pI but with decreasing molecular weights) is typically observed for *T. spiralis *E-S proteins (Bien, data not shown), and we suspect that these protein spots could result from post-translational modification presumably resulting from proteolysis. Proteolysis has been proven to be responsible for post-translational modification of secreted *T. spiralis *E-S protein called MCD-1 (multi-cystatin-like domain protein 1) [51]. MCD-1 belongs to family of cystatins, which exist as high- and low- molecular weight isoforms formed by alternative mRNA splicing, and the resulting proteins are then proteolytically processed to release bioactive peptides [52]. However, there is no evidence of the role of alternative splicing in production of MCD-1 isomers [51]. Instead, the formation of low-molecular weight MCD-1 isomer is suggested to result from proteolytic cleavage of the C-terminal domain III from the secreted protein [51].

Identifications related to proteins showing differential abundance included 10 protein spots from which 5 protein spots (spots 1, 33, 37, 46, 52) were more abundant in *T. britovi *and 5 (spots 5, 30, 31, 45, 65) were more abundant in the *T. spiralis *E-S proteome. In *T. britovi*, these proteins were identified as different glycoproteins (gp43, p49) and serine-protease isoforms as well as a 5'-nucleotidase (Additional file 1: Table S2). These proteins were identified also from *T. spiralis*, suggesting that different forms of the same E-S proteins are more produced by this species (Additional file 1: Table S2). Thus, we suggest that both species produce equivalent E-S proteins, which are post-translationally modified to a different extent, which in certain cases seemed to result in species-specific modification. It is tempting to speculate that such differences could contribute to the mechanism by which these two species select the host and establish successful infection.

### Immunoproteomic analysis of *T. spiralis *and *T. britovi *E-S proteins

An immunoproteomic approach was next used to verify which of the identified proteins cross-react with species-specific antisera. For this purpose, proteins from E-S extracts of *T. spiralis *and *T. britovi *were separated by 2-DE and transferred onto PVDF membrane for treatment with sera from pigs experimentally infected with *T. spiralis *or *T. britovi*. The experiments, conducted in triplicate, were highly reproducible, yielding similar patterns of immunoreactive proteins (Figure 2B). As shown in Figure 2B, the 2-D immunoblotting of *T. spiralis*- and *T. britovi *E-S proteins with specific antisera results in somewhat different immunoproteomes, including both specifically (spots 5, 9, 46, and 31, 45, 50) and commonly (spots 19, 37, 52) recognized antigens. In *T. britovi*, the cross-reactive E-S proteins (10 in total) were found to migrate with pI values around 6 and with molecular weight ranging from 40 to 50 kDa. In the case of *T. spiralis*, proteins (10-12 in total) detected with antisera were found to migrate at 40 kDa with slightly higher pI values than those detected with anti-*T. britovi *antibodies (Figure 2B).

A previous 2-D immunoblotting study by Wu et al., demonstrated that the E-S products of *T. spiralis *have mainly three kinds of antigenic proteins composed of 5-11 isoforms that migrate at about 40-50 kDa [53]. These findings were supported by another study that demonstrated a similar antigenic pattern for the *T. spiralis *E-S proteins, with three major protein bands migrating between 40-60 kDa that showed high cross-reactivity [54]. In our study, the antigenic proteins had a lower molecular mass, which could be a result of the methods used for proteome analyses and/or individual variations between the antisera used in the present and in the published studies. In the case of *T. britovi*, 2-D immunoblotting revealed two groups of horizontally adjacent antigenic spots (Figure 2B). Previous 1-DE and 2-DE immunoblotting studies involving the crude extract isolated from *T. britovi *and *Trichinella *genotype 8 ML revealed that the major antigens are acidic with molecular mass values ranging from 45 to 70 kDa [35,54]. These highly cross-reactive proteins of *T. spiralis *and *T. britovi *have been identified as glycoproteins with a postulated role in infection and nurse cell development [55,56]. Moreover, we suspected that the most prominent E-S proteins that were found to react with pig antisera likely corresponded to those found by Robinson and Connolly [36] and could be modified by tyvelose, the dominant and highly immunogenic sugar found in both the TSL-1 antigens and E-S antigens [46]. The tyvelose bearing proteins present in E-S products have been suggested to play a role in establishment and maintenance of the nurse cell, and play a role as mediators in the intestinal phase [57-59]. These proteins may also induce a powerful antibody response in parasitized animals and can be used for immunodiagnostic purposes [60].

In the present study, the specific antibodies against *T. britovi *were found to cross-react with protein spots matching to the location of different 5'-nucleotidase and serine-protease isoforms in the 2-DE-gel (Figure 2A), while the specific antibodies against *T. spiralis *recognized only certain isoforms of the gp43 glycoprotein and serine-proteases. Interestingly, the cell-surface display of the 210-239 amino acid epitope derived from gp43 of *T. spiralis *ML on *Salmonella *was recently shown to induce a protective immune response against the *T. spiralis *challenge in mice [61]. Proteins specifically recognized by *T. britovi *antisera only included the different 5'-nucleotidase isoforms. Interestingly, one of the protein spots (spot 5), representing one of the 5'-nucleotidase variants, is produced less by *T. britovi *ML compared to *T. spiralis *(Figure 3A). This finding suggests that this particular form of the 5'-nucleotidase, which is only recognized by *T. britovi *antisera, is highly antigenic. It is tempting to speculate that differential expression of 5'-nucleotidase contributes to immune evasion since it is currently believed that this enzyme is able to regulate host immune and inflammatory responses by modulating nucleotide levels during infection [62].

Interestingly, both antisera also recognized common proteins, such as those identified from spots 19, 37 and 52 (gp43, serine protease/proteinase). This finding indicates that these proteins are more efficiently recognized by the specific antibodies against *T. spiralis*, since according to 2-D DIGE the corresponding protein spots are more abundant in *T. britovi *compared to those detected in *T. spiralis *E-S proteome (Additional file 1: Table S2, Figure 3B). Both of these proteins have been previously shown to be stage-specific ML proteins; the cross-reacting gp43 possess a DNase II-like motif and is suggested to be involved in the formation of nurse cells, while the identified protease is believed to play a crucial role in the development or migration of NBL in host tissues [63]. In addition, proteinase can also serve as an immunodominant antigen, stimulating a protective immune response [64]. Previous studies have demonstrated that *T. spiralis *ML antigens fall into eight groups according to their recognition by monoclonal and/or polyclonal antibodies. Furthermore, monoclonal antibodies raised against *T. spiralis *and *T. pseudospiralis *also recognize *T. britovi *[65,66]. In the present study, the 2-DE immunoblotting analyses strengthen these findings by showing that both *T. spiralis *and *T. britovi *antisera recognize a common set of antigens, whereas *T. britovi *antisera also cross-reacted with several isoforms of the 5'-nucleotidase.

## Conclusions

Early clinical diagnosis of trichinellosis is likely to contribute to improved treatment outcomes, however the differentiation of causative species, by serological or biochemical means, has remained proven difficult. In the present study we applied 2-D DIGE and 2-D immunblotting to screen for immune-reactive E-S antigens of diagnostic potential from two important *Trichinella *species, *T. spiralis *and *T. britovi*. In this regard, 17 proteins exhibiting differential expression were identified by LC-MS/MS. The identified proteins included 5'-nucleotidase, serine-type proteinase/protease, gp43 and p49 antigens; some of which were suggested to have undergone charged post-translational modification that, in certain cases, seemed to result in species-specific modification of the protein. The antigenic differences of the *Trichinella *species analyzed by 2-D immunoblotting demonstrated that, in addition to recognizing the same proteins, specific antibodies against *T. britovi *also specifically recognized the different isoforms of the 5'-nucleotidase. To the best of our knowledge, this is the first proteomic study to focus on *T. britovi *E-S proteins and to show specific immunoreactive proteins among the E-S products of this species.

## Abbreviations

2-DE: Two-dimensional electrophoresis; 2-D DIGE: Two-dimensional difference gel electrophoresis; LC-MS/MS: Liquid chromatography-tandem mass spectrometry; E-S: Excretory-secretory; IEF: Isoelectric focusing; ML: Muscle larvae; DIA: Differential in-gel analysis; BVA: Biological variation analysis; NBL: Newborn larvae; PVDF: Polyvinylidene difluoride.

## Competing interests

The authors declare that they have no competing interests.

## Authors' contributions

JB, AN, KG and KS conceived the study idea, designed it and performed the most of the experiments. JB, KS and AN prepared the first draft of the manuscript. JB and KG prepared the antigen proteins. JB, KS and PV performed the 2-D DIGE experiments and immunoblotting. KS and TAN did mass spectrometric analyses. KS and PV helped with the data analysis and interpretation. BM and WC did the theoretical supervising of the manuscript. All the authors read and approved the final manuscript.

## Supplementary Material

Additional file 1 **Table S2**. Identification of *T. britovi *and *T. spiralis *E-S proteins showing differential abundance in 2-D DIGE gels. The grey or white backgrounds correspond to proteins that are produced more in *T. britovi *or *T. spiralis*, respectively. Click here for file
