# Supplementary material for: Comparative analysis of excretory-secretory antigens of Trichinella spiralis and Trichinella britovi muscle larvae by two-dimensional difference gel electrophoresis and immunoblotting
Source: Proteome Sci. 2012 Feb 11;10:10. doi: 10.1186/1477-5956-10-10 (PMC3305586; doi:10.1186/1477-5956-10-10)
Supplement: Additional file 1 — Table S2. Identification of T. britovi and T. spiralis E-S proteins showing differential abundance in 2-D DIGE gels. The grey or white backgrounds correspond to proteins that are produced more in T. britovi or T. spiralis, respectively. [file 1477-5956-10-10-S1.DOC]

**Table 2. Identification of *T. britovi* and *T. spiralis* E-S proteins showing differential abundance in 2-D DIGE gels. The grey or white backgrounds correspond to proteins that are  produced more in *T. britovi* or *T. spiralis*, respectively.**

| **Spot**  **no.^a^** | **Name of protein** | **Access. no. closest homologue/ Strain^b^** | **Appear.^c^** | ***p*-value^d^** | **Av. vol. ratio^e^** | **Theoretical^f^**  **Mw pI** | | **[ms] score^g^** | **No. matched**  **peptides^h^** |
| --- | --- | --- | --- | --- | --- | --- | --- | --- | --- |
| 1 | 49 kDa E-S protein  p49 antigen | AY486427/*T. nativa*  AAA30328/ *T. spiralis* | 12(12)  12(12) | 2,60E-08  2,60E-08 | 14.84  14.84 | 34.8  34.5 | 5.12  5.23 | 294  276 | 9  9 |
| 2, 9, 22, 46 | 5’-nucleotidase  5’-nucleotidase  Ecto-5’-nucleotidase | AY127571/ *T. spiralis*  AAM97494/ *T. spiralis*  XP_003380307/ *T. spiralis* | 12(12)  12(12)  12(12) | 4.90E-05 – 5.50E-08 | 3.78 –14.84 | 62.4  62.3  65.8 | 6.13  6.13  5.62 | 195-495 | 14-18 |
| 5 | Secreted 5’-nucleotidase | AY127571/ *T. spiralis* | 12(12) | 5,30E-07 | 1.54 | 62.4 | 6.13 | 495 | 14 |
| 4 | -**^j^** | - | 12(12) | 2.20E-07 | 27.04 | - | - | - | - |
| 6 | -**^j^** | - | 12(12) | 6.10E-07 | 65.53 | - | - | - | - |
| 19 | Serine proteinase  Serine protease  Putative trypsin  p49 antigen  p43 glycoprotein | AY028974/ *T. spiralis*  ACA28932/ *T. spiralis*  XP_003376874/*T. spiralis*  AAA30328/ *T. spiralis*  AAA30327/ *T. spiralis* | 9 (12)  9(12)  9(12)  9(12)  9(12) | 3.40E-08  3.40E-08  3.40E-08  3.40E-08  3.40E-08 | 14.03  14.03  14.03  14.03  17.26 | 48.7  48.7  53.9  34.5  38.1 | 6.33  6.33  5.97  5.23  5.96 | 167  167  167  111  111 | 16  16  16  3  3 |
| 24 | 49 kDa E-S protein | AY486427/ *T. nativa* | 12(12) | 6.00E-06 | 14.75 | 34.8 | 5.12 | 220 | 5 |
| 30 | Serine proteinase  Serine proteinase | AY028974/ *T. spiralis*  ACA28930/ *T. spiralis* | 12(12)  12(12) | 9.50E-06  9.50E-06 | 3.65  3.65 | 48.7  51.3 | 6.33  6.25 | 264  80 | 10  3 |
| 31 | -**^j^** | - | 12(12) | 1.20E-05 | 4.58 | - | - | - | - |
| 32 | P49 antigen  49 kDa E-S protein  43 kDa glycoprotein | AAA30328/ *T. spiralis*  AAR83176/ *T. nativa* AAA30327/ *T. spiralis* | 12(12)  12(12)  12(12) | 1.50E-05  1.50E-05  1.50E-05 | 17.26  17.26  17.26 | 34.5  34.8  38.1 | 5.23  5.12  5.95 | 51  51  51 | 1  1  1 |
| 33 | Serine proteinase  p49 antigen  Serine proteinase SP-1 | AY028974/ *T. spiralis*  AAA30328/ *T. spiralis*  ABY73337/ *T. pseudospiralis* | 12(12)  12(12)  12(12) | 1.80E-05  1.80E-05  1.80E-05 | 3.29  3.29  3.29 | 48.7  51.5  34.5 | 6.33  6.07  5.23 | 93  84  81 | 6  3  3 |
| 37 | Serine proteinase | AY028974/ *T. spiralis* | 12(12) | 2.90E-05 | 4.27 | 48.7 | 6.33 | 84 | 5 |
| 40 | -**^j^** | - | 12(12) | 3.40E-05 | 28.33 | - | - | - | - |
| 41 | -**^j^** | - | 12(12) | 3.50E-05 | 26.27 | - | - | - | - |
| 45 | 43 kDa secreted glycoprotein | AAA30327/ *T. spiralis* | 12(12) | 4.70E-05 | 9.62 | 38.1 | 5.95 | 161 | 4 |
| 50 | Serine proteinase | AY028974/ *T. spiralis* | 12(12) | 0.00012 | 5.57 | 35.7 | 5.97 | 40 | 1 |
| 52 | Serine protease | AAK31787*/ T. spiralis* | 12(12) | 0.00018 | 4.3 | 48.7 | 6.33 | 62 | 3 |
| 60 | p49 antigen**^k^**  49 kDa E-S protein | M64242/ *T. spiralis*  AAR83176/ *T. nativa* | 12(12)  12(12) | 0.00078  0.00078 | 5.49  5.49 | 34.3  34.8 | 5.23  5.12 | 66  66 | 1  1 |
| 65 | Putative trypsin**^i^** | XP_003381667/ *T. spiralis* | 12(12) | 0.0092 | 2.83 | 72.8 | 8.83 | 717 | 21 |

**^a)^** Spot numbers correspond to those in Figure 1.

**^b)^** Accession number of the closest homologue obtained from the NCBI database using the identified protein as the query sequence.

**^c)^** Number of times spot was detected from a total of 12 images.

**^d)^** Calculated from *t*-test.

**^e)^** The average volume value ratio based on the normalized spot volume standardized against the intragel standard provided by

DeCyder software analysis.

**^f)^** Theoretical MW (kDa) and p*I* values were obtained from MASCOT search results.

**^g)^** Mascot Mowse propability score (p < 0.05).

**^h)^** Number of matched mass values.

**^i)^** Evaluation of the identified peptides suggest that this protein has undergone proteolytic processing, thus the observed pI and Mw values are suggested to correspond to the identified trypsin after proteolytic processing.

**^j)^** Protein could not be identified.

**^k)^** The native 49 kDa antigen has been shown to be a glycosylated protein (46-48), therefore the observed pI and Mw values are suggested to correspond to the glycosylated form of the protein.
